# Overview of follicle stimulating hormone and its receptors in reproduction and in stem cells and cancer stem cells

**DOI:** 10.7150/ijbs.63721

**Published:** 2022-01-01

**Authors:** Swati Haldar, Himanshu Agrawal, Sarama Saha, Alex R. Straughn, Partha Roy, Sham S. Kakar

**Affiliations:** 1 Molecular Endocrinology Laboratory, Department of Biosciences and Bioengineering, Indian Institute of Technology Roorkee, Uttarakhand 247667, India.; 2 Current address: Drug Discovery and Development Division, Patanjali Research Institute, Haridwar, Uttarakhand 249405.; 3 Department of Biochemistry, All India Institute of Medical Sciences Rishikesh, Uttarakhand 249203, India.; 4 Department of Physiology, James Graham Brown Cancer Center, University of Louisville, Louisville, KY 40202, USA.

## Abstract

Follicle stimulating hormone (FSH) and its receptor (FSHR) have been reported to be responsible for several physiological functions and cancers. The responsiveness of stem cells and cancer stem cells towards the FSH-FSHR system make the function of FSH and its receptors more interesting in the context of cancer biology. This review is comprised of comprehensive information on FSH-FSHR signaling in normal physiology, gonadal stem cells, cancer cells, and potential options of utilizing FSH-FSHR system as an anti-cancer therapeutic target.

## Introduction

**Figure 1:**
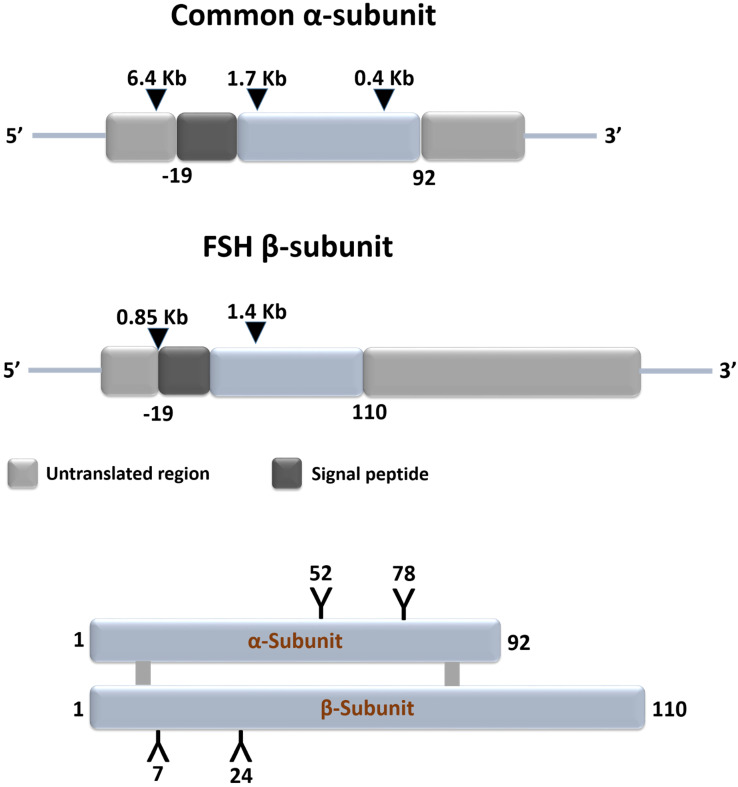
** Schematic representation of structures of the common subunit (α-) and FSHβ genes (upper panel) and of FSH protein (lower panel).** The symbols 'Y' in the lower panel marks the approximate positions of the N-linked carbohydrate side chains in the FSH molecules. Adapted from Huhtaniemi and Aittomaki 14.

Gonadotrophs of the anterior pituitary gland synthesize and secrete follicle stimulating hormone (FSH) in response to stimulation by the hypothalamic gonadotropin releasing hormone [GnRH also referred to as luteinizing hormone releasing hormone (LHRH)] 1. FSH is a glycoprotein and plays an important role in: growth, development, pubertal maturation, and reproductive physiology 2,3. It is a 35.5 kDa heterodimeric protein that shares structural similarity with other glycoprotein hormones of pituitary and placental origin such as luteinizing hormone (LH), thyroid-stimulating hormone (TSH), and human choriogonadotropin hormone (hCG) 1,4. FSH has a functionally indispensable 96 amino acid alpha- (α-) subunit that is common to LH, TSH and hCG, in addition to a structurally unique beta- (β-) subunit 5,6 (Figure 1).

Functional specificity of FSH is provided by its β-subunit that ensures FSH interaction specifically with FSH receptor (FSHR) 7. However, some computational and crystallographic studies have noted the interaction of the α-subunit with FSHR, suggesting that both α- and β- subunits interact with surface receptors 8. The constituting oligosaccharides, namely: N-acetylglucosamine, galactose, N-acetylgalactosamine, mannose, and sialic acid, are attached to both subunits by covalent bonding via asparagine through N-linked glycosylation 9,10. Glycosylation facilitates hormonal participation in protein folding, dimer stability, and secretion process, in addition to influencing metabolic clearance and receptor activation 11. Like other glycoproteins, sialic acid heterogeneity leads to variations in glycoprotein composition and differences in the isoelectric charge of FSHR. Consequently, FSHR displays various chemical isoforms due to both structural variations and differences in charge. These glycoforms have been shown to have differential functional effects in *in vitro* studies. In an earlier study, Timosi et al. 12 demonstrated that different isoforms of FSHR function distinctly in rat granulosa cells. Seven isoforms that vary in sialic acid content were purified from the pooled anterior pituitary extracts with the help of high resolution chromatofocusing and affinity chromatography and analyzed for their potential to impact granulosa cells function during culture conditions. Less acidic FSHR isoforms induced the cAMP release, cytochrome P450 aromatase mRNA expression, oestrogen synthesis and tissue type plasminogen activity (tPA) more strongly than more acidic form. FSHR isoform with high acidic content was found to be more potent in stimulating the mRNA synthesis of α-subunit of inhibin 12. Recently, Loreti et al. 13 also found that different human FSHR sialic acid variants affect the endocrine activity and global gene expression in human ovarian granulosa like tumor cell line- KGN 13. Less sialyated recombinant human FSHR were found to be more potent in stimulating the synthesis of oestradiol, progesterone and inhibin A. Similar to these observations, microarray data analysis also revealed that level of hormone sialyation differentially affected the expression of various gene relevant to the function of granulosa cells.

**Figure 2:**
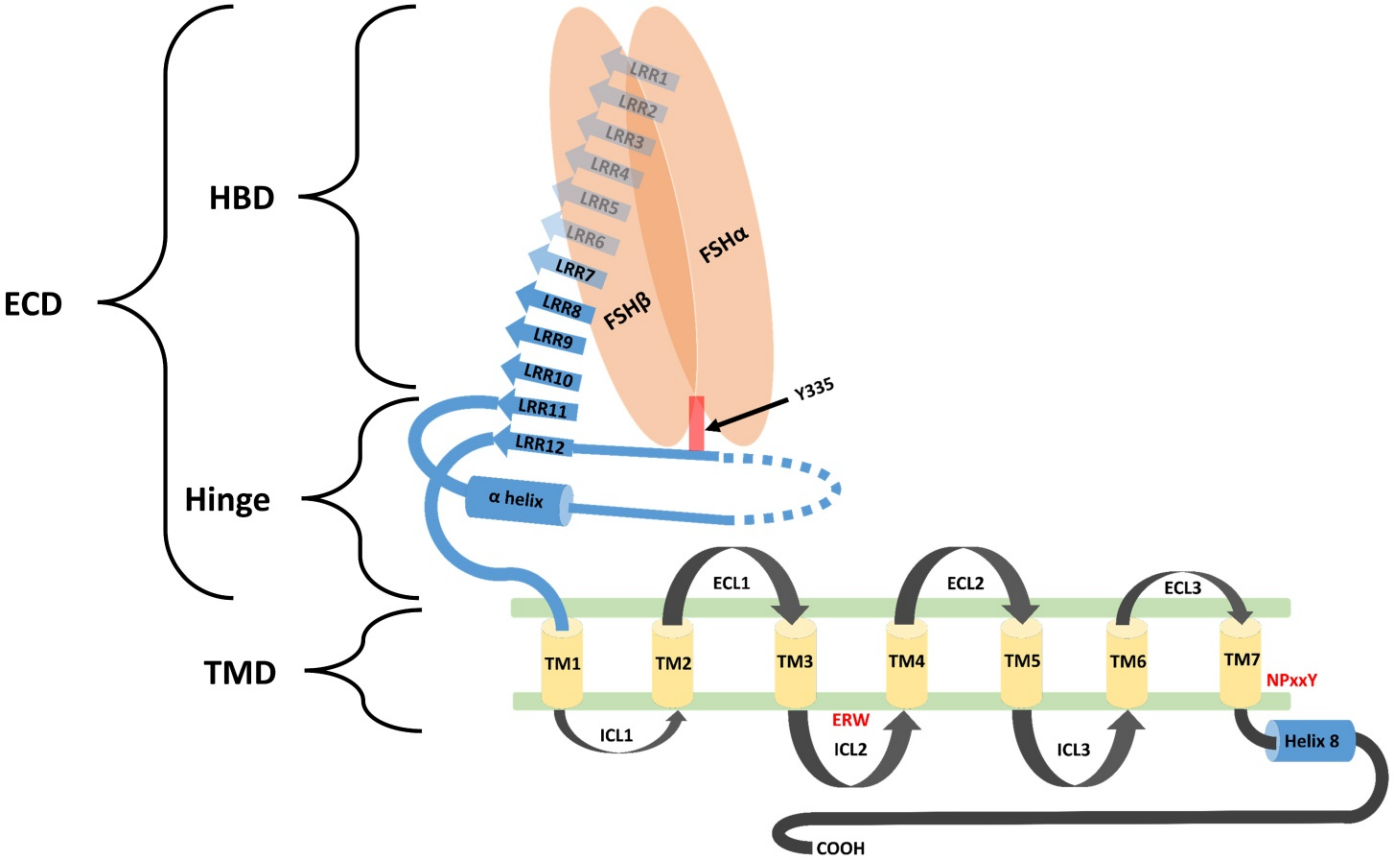
** Schematic diagram representing the structural component of FSHR1.** FSHR1 structure consist of an N-terminal extracellular domain, transmembrane domains and intracellular domains. Extracellular domain may be further divided into two subdomains, hormone binding domain (HBD) and hinge region. HBD comprises the ten 10 consecutive leucine rich repeats (LRR). Hinge region covers two other LRR, a hair pin loop and an alpha helix. A sulphated tyrosine at the 335^th^ amino acid position of a hinge loop in the signal-specificity sub-domain is responsible for the hormonal activity. A short intra-cellular domain rich in serine and threonine molecules serve as a potential phosphorylation sites during signal transduction. Leucine rich repeats are demonstrated by the blue colored arrows. The α-Helix in the hinge region is shown as a blue colored cylinder. Transmembrane helices (1-7) are depicted as yellow colored boxes. FSH-α and FSH-β subunits are demonstrated by orange ovoid shapes. ECL: Extracellular loop; ICL: Intracellular loop; ECD: Extracellular domain; TMD: transmembrane domain; HBD: Hormone binding domain. Re-drawn from Figure 2 of Pascali et al. 17.

Biological functions of FSH are activated through its interaction with its specific receptors (i.e. FSHR1). Human FSHR1 protein is composed of 695 amino acids polypeptide including the signal peptide. A mature FSHR1 protein of 76 kDa molecular mass is expressed in Sertoli and granulosa cells 15. Since FSHR1 possesses seven transmembrane helices, therefore it is also known as 7-Trans Membrane Receptor (7TMR). It consists of an N-terminal extracellular domain, 7-transmembrane domians, 3-extracellular loops, 3-intracellular loops and a C-terminal intra-cellular domain. The glycosylated extracellular domain has twelve leucine-rich repeats and consists of hormone-binding and signal-specificity sub-domains. A sulphated tyrosine at the 335^th^ amino acid position of a hinge loop in the signal-specificity sub-domain is responsible for the hormonal activity 7. Disulfide bonds between highly conserved cysteine residues of the transmembrane domain stabilize the structure of FSHR1. The highly conserved Asp-Arg-Tyr triplet in GPCRs is believed to be responsible for signal transduction. However, in FSHR1, a Glu-Arg-Trp triplet is a variant of the highly conserved Asp-Arg-Tyr sequence normally found in GPCRs, which is believed to be responsible for signal transduction 16. The transmembrane domain is followed by a short intra-cellular domain, which is rich in serine and threonine molecules that serve as potential phosphorylation sites during signal transduction. A comprehensive structure of FSH-FSHR1 is depicted in Figure 2.

### Different isoforms of follicle stimutating hormone receptor (FSHR)

In humans, the FSHR gene spans 52 Kbp on chromosome 2 and consists of eleven exons and ten introns. FSHR gene exhibits four alternatively spliced forms (i.e. FSHR1, FSHR2, FSHR3 and FSHR4). Among these spliced forms, FSHR1 and FSHR3 are more recognized for their participation in various functions of FSH, including: cell growth, proliferation, and steroidogenesis 18,19. Unlike FSH, which consists of a common α-subunit and a specific β-subunit, FSHR has different isoforms, implicating a diverse range of functions to be under its signaling influence. Therefore, a brief discussion on the functionalities of each of these FSHR isoforms in normal tissues is pertinent prior to coursing into the metabolic effects of their malfunctions. The remaining portion of this section has been dedicated towards understanding the functions performed by each of the four identified FSHR isoforms.

**Figure 3:**
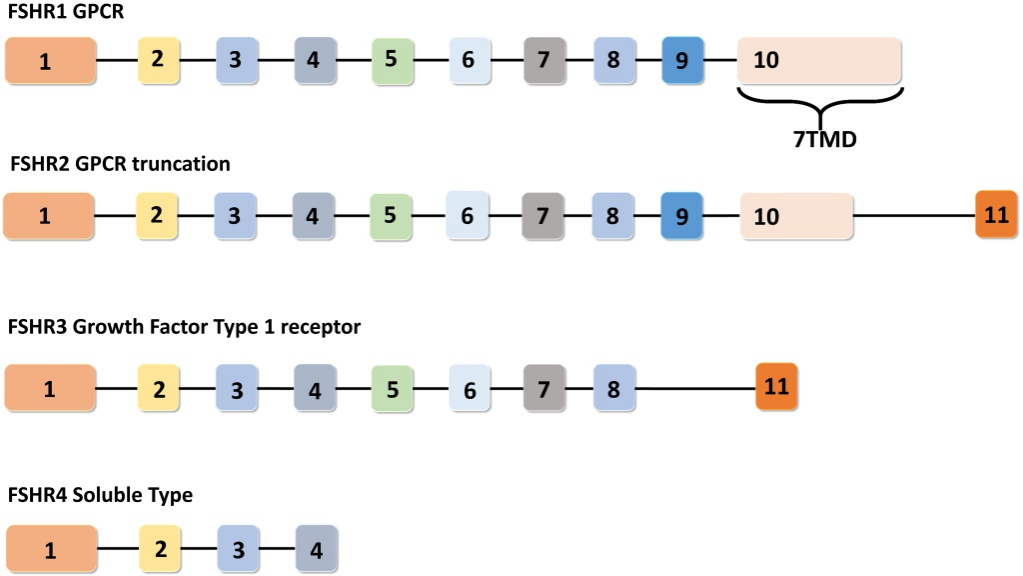
** Exon structures of FSHR splice variants.** Canonical FSHR1 (GPCR) contains 10 exons, with the 7 transmembrane domains encoded by exon number 10. FSHR2 (dominant negative isoform) is the truncated form of FSHR1, where exon 10 is truncated and joined with exon number 11. In FSHR3 (Growth factor type 1 receptor), exons 9 and 10 are spliced out and the transcript is joined to exon 11. FSHR4 is considered to be a soluble-type receptor and only contains exons 1-4. Adapted from Bhartiya and Singh 19.

Among the four alternatively spliced isoforms of FSH receptors, FSHR1 transcript contains all the ten exons and is known as the major isoform that actively participates in steroidogenesis, follicular development, and spermatogenesis. It has a large N-terminal extracellular domain, seven α-helical transmembrane domains interconnected with alternating extracellular and intracellular loops, and an intracellular C-terminal tail 17. The FSHR2 variant is structurally different from the FSHR1 splice variant, as evidenced by the deletion of the entire intracellular domain and a portion of the transmembrane domain. The FSHR2 transcript contains a truncated exon 10, which is then joined to exon 11 (normally excised in FSHR1 transcript). Therefore, FSHR2 contains the extracellular domain and a portion of the transmembrane domain found in FSHR1 19,20. FSHR3 possesses an extracellular domain and a single transmembrane domain. In the FSHR3 transcript, exons 9 and 10 are spliced out, leaving exons 1-8 joined with exon 11 21. With only one transmembrane domain, FSHR3 is structurally different from both the FSHR1 and FSHR2 isoforms, with its overall topology more akin to the growth factor type I receptor. FSHR4 is dissimilar to all the three isoforms and exists as a soluble form of the receptor without any transmembrane domain. The exon structures of different splice variants of FSHR are depicted in Figure 3.

FSHR1 is a member of the G-protein-coupled receptors (GPCRs) family and activates the G-protein (Gs), resulting in the production of cyclic adenosine monophosphate (cAMP), which subsequently leads to the induction of various signaling pathways after the initial binding to FSH 20,22. G-protein coupling to the FSH-FSHR1 complex is mediated by the intracellular domains of the receptor. These domains are also responsible for the induction of downstream signaling cascades. The cAMP/protein kinase A (PKA)/cAMP response element-binding protein (CREB) pathway is one of the most prominent pathways activated by the FSH-FSHR1 complex, which is responsible for expression of the genes responsible for the proper functioning of granulosa cells, like aromatase and inhibin-α, among others 23. With the activation of cAMP/exchange protein, the cAMP/RAS-related protein 1 (RAP1) pathway is induced, leading to activation of the downstream phosphoinositide 3-kinase (PI3K) pathway 24,25. Additionally, the FSH-FSHR1 interaction activates several kinases, such as: Src family kinases (SFKs), mitogen-activated protein kinases (MAPKs), and extracellular signal-regulated kinases (ERKs), which are the key factors in some of the crucial metabolic pathways 20,26. The FSH-FSHR1 complex, in conjunction with epidermal growth factor receptor (EGFR), regulates RAS and ERK1/2 pathways 23. From the information obtained from preclinical studies, the FSH-FSHR1 pathway has been associated with increased angiogenic potential of granulosa cells of the ovary, conducive for follicular maturation. It has been noted that the FSH-FSHR1 complex facilitates secretion of pre-angiogenic factors, such as platelet-derived growth factor (PDGF)-β and vascular endothelial growth factor (VEGF) from granulosa cells, through transforming growth factor (TGF)-β1 27. Although the effect of the FSH-FSHR1 pathway on VEGF expression requires more clarity, this initial observation is suggestive of the involvement of FSH-FSHR1 interaction in vascular development in most, if not all rapidly growing cell populations 28.

FSHR2 is a truncated form of FSH receptor and this truncation disrupts the intracellular portion of the receptor and negatively affects the downstream signaling process 21. Although, FSHR2 binds FSH with a high affinity, however it fails to induce G-protein coupled signaling. As a result, it is referred as dominant negative receptor 29-31. Failure to activate the usual G-protein-mediated signaling pathway observed in response to FSHR1 is attributed to the binding of FSHR2 to the inhibitory G_i_, instead of the stimulatory G_αs_ protein. Further research remains to be performed to understand the physiological significance of the FSHR2 splice variant. FSHR3 possesses a distinguishing feature from FSHR1. It functions independently of cAMP-controlled pathways 32. FSHR3 exhibits a growth factor type properties by actively participating in the mitotic activity and cell proliferation in its target cells. FSHR3 activates the mitogen-activated protein kinase-extracellular signal-regulated kinase (MAPK-ERK) pathway in granulosa cells in a cAMP-independent manner 31-33. The C-terminal portion of FSHR3 is equipped with a consensus sequence cognate for phosphorylation by MAPK 18,34. FSHR3 mediated activation of MAPK-ERK pathway regulates the cell proliferation through calcium ion (Ca^2+^) influx via modulation of Ca^2+^-dependent channels 34,35. Proliferation of ovarian surface epithelial cells in response to FSHR3 mediated MAPK activation further confirms its mitogenic and proliferative functions 35,36. The function of FSHR4 is not presently defined, although it is assumed that FSHR4 binds with FSH within the extracellular matrix to prevent the latter from binding to any of the other three FSHRs, a *modus operandi* similar to that of insulin-like growth factor (IGF)-1-binding proteins 37. FSHR4 has also been suggested as a prohormone of the active molecule 36. Expression of different FSHR isoforms indicates more than one pathway being modulated by FSH signaling. Therefore, multi-cascade functions from cell proliferation to cell differentiation by FSH-FSHR signaling can partly be attributed to the involvement of different variants of FSHRs 38.

### Role of FSH-FSHR signalling in stem cell modulation

Existing literature suggests the presence of spermatogonial stem cells (SSCs) in the mammalian testis. Further, recent reports demonstrate that stem cells are localized in adult ovaries and undergo postnatal oogenesis, similar to spermatogenesis in the testis 39,40. Concurrently, a minor population of very small embryonic stem cells (VSELs) have also been observed along with SSCs in the testis. These cells have been shown to express FSHR and respond directly to FSH 41,42. In the following section, we enumerate on the current information regarding the role of FSH-FSHR signaling in ovarian and testicular stem cell.

### Function of FSH-FSHR signaling in ovarian stem cells

In reproductive biology, a general dogma exists which states that the mammalian ovary contains a fixed number of follicles that are depleted progressively with the advancement of age and culminates in menopause. However, this belief has been challenged by the existence of stem cells in adult ovaries. Various groups have demonstrated the presence of ovarian stem cells in adult mammalian ovary and put forth a hypothesis in favor of ovarian stem cells (OSCs) for their role in neo-oogenesis and primordial follicular (PF) assembly, similar to that of SSCs in spermatogenesis. According to this hypothesis, the presence of OSCs leads to the regular assembly of follicles on ovarian surface epithelial/cortical regions and that the process of primordial follicle assembly from stem cells is regulated by FSH 43.

Menopause probably occurs due to an age-related compromise in the somatic niche and, under this situation, OSCs are unable to undergo differentiation, thus leading to menopause 44. Despite a unanimous consensus on the existence of OSCs in adult ovaries, strong evidence in support of their function in neo-oogenesis and follicular assembly under *in vivo* conditions needs to be developed. Recent studies have demonstrated the localization of stem cells in adult mammalian ovary surface epithelium. The very first study that challenged the concept of a fixed reserve of follicles in females was reported by Tilly and his group 45 showing the presence of 5-8 μm OSCs in mouse ovarian surface epithelium (OSE). Consistent with this information, Virant-Klun's group demonstrated the existence of human embryonic stem cell marker, SSEA-4 positive small stem cells (3-5 μm) that express various markers related to pluripotency and primordial germ cells 46. Further, the same group reported the spontaneous differentiation of these stem cells into oocyte-like structure under *in vitro* conditions 47. However, this concept of stem cells in adult ovaries is still not widely accepted and encounters certain technical challenges 19. Stem cell populations have been isolated from different species, including: mouse, sheep, human, rabbit, and monkey, through gentle scraping of OSE. They are divided into two categories based upon the localization of the pluripotent marker protein OCT-4 48-51. VSELs expressed nuclear OCT-4, while slightly bigger OSCs showed a cytoplasmic localization of OCT-4. VSELs isolated from gonads spontaneously differentiated into the sperm and oocytes *in vitro* without any specific requirement of growth factor/ cytokine and thought to be developmentally equivalent of primordial germ cells - the natural precursor of gametes 52-54. VSELs have a high nucleocytoplasmic ratio, possess a capacity for self-renewal and could differentiate into OSCs 44,50. In contrast, OSCs were reported to undergo rapid cell division and formed germ cell nest in response to FSH treatment 19,50,55. Treatment of mice with pregnant mare serum gonadotropin (PMSG, an FSH analogue) stimulated the proliferation of OSE, which promoted the assembling of fresh follicular cells beneath the OSE 55.

Both FSHR1 and FSHR3 were found to be expressed in sheep OSCs, whereas the epithelial cells were distinctly negative for any type of FSHR. An active transcription of FSHR3 was observed in OSCs in response to FSH within 3 h, although FSHR1 levels remained unaffected 51. Patel *et al.* 56 observed that OSCs in sheep respond to FSH through FSHR3 and as a result of this interaction, OSCs experienced proliferation and clonal expansion that led to the formation of a germ cell nest, which could differentiate into oocytes 56. Since VSELs are quiescent in nature, Sriraman et al. 57 examined whether VSELs can survive chemotherapy treatment and examined their potential to initiate neo-oogenesis after treatment with FSH. Treatment of chemoablated ovary with PMSG for 48 h was found to significantly increase the number of VSELs. A similar effect of FSH treatment was also observed in mechanically isolated OSEs from the chemoablated ovary. OSE culture in the presence of FSH resulted in the formation of PCNA and OCT-4 positive germ cell nests and further supported the formation of oocyte-like structure 57. In contrast, some studies reported the absence of stem cells in mouse ovaries and proposed that primordial follicles developed during fetal life are appropriate for oogenesis and that oocyte renewal does not occur during the adult life 58. Similarly, Zhang *et al.* 59 also reported the absence of post-natal oogenesis using a genetically modified mouse model. Nevertheless, this continues to be a debatable issue that requires extensive exploration and contextual evidence.

The evidences prove the role of FSH in stimulating OSCs. Nevertheless, direct action of FSH on ovarian stem cells and its physiologic function can be demonstrated only through knockout/down and overexpression studies on FSHR. To the best of our knowledge, there is no study that deals with the knockout/down of FSH/FSHR to establish the physiological role of FSH/FSHR in ovarian stem cells. However, the results obtained from earlier animal knock down studies of FSH/FSHR clearly demonstrate the obligatory role of FSH-FSHR in the female reproductive system 60. Two groups have examined the effect of knockout of FSHR gene in mice 61,62. These reports demonstrate that as expected FSHR-/- female mice were sterile with a markedly reduced level of estradiol and progesterone. Female FSHR-/- mice had a small size of uterus and impaired follicular maturation with absence of mature follicle. Furthermore, the size of several reproductive organs such as uterine, ovarian and vagina were also reduced. Nevertheless, knock-out studies directly establishing FSH action on OSCs are still awaited. However, gain-of-function *in vivo* models to understand FSH functioning in physiological context contributed significantly towards our understanding of the modus operandi of this critical molecular partnership 63.

### Function of FSH-FSHR system in testicular stem cells

The process of spermatogenesis originates from SSCs and involves several sequential steps of cell proliferation and differentiation, which lead to the development of spermatozoa 64. In addition, interaction of several growth factors and hormones with germ cells and Sertoli cells regulates the process of spermatogenesis that ultimately leads to the generation of a functional spermatozoa 65. According to classical knowledge, two pituitary hormones, LH and FSH, play a significant role in spermatogenesis. LH stimulate the production and secretion of testosterone from the Leydig cells present in the interstitial space in the testis; whereas FSH either independently or in association with LH promotes the proliferation of Sertoli cells and facilitates the synthesis of various signaling molecules and nutrients that assist the initiation and maturation of functional spermatid.

Conversely, testosterone interacts with androgen receptors in Sertoli cells and regulates several responses involved in spermatogenesis 66,67. *In vitro* studies have been conducted to delineate the role of FSH in the development of Sertoli stem cells. Both *in vitro* and *in vivo* studies suggest that FSH acts as an important mitogenic factor for Sertoli cells and stimulates their proliferation. Additionally, FSH also stimulates the synthesis of certain growth factors, like Glial cell line-derived neurotrophic factor (GDNF) and fibroblast growth factor 2 (bFGF2), in Sertoli cells that supports the proliferation and colonization by type A SSCs 68,69.

**Figure 4:**
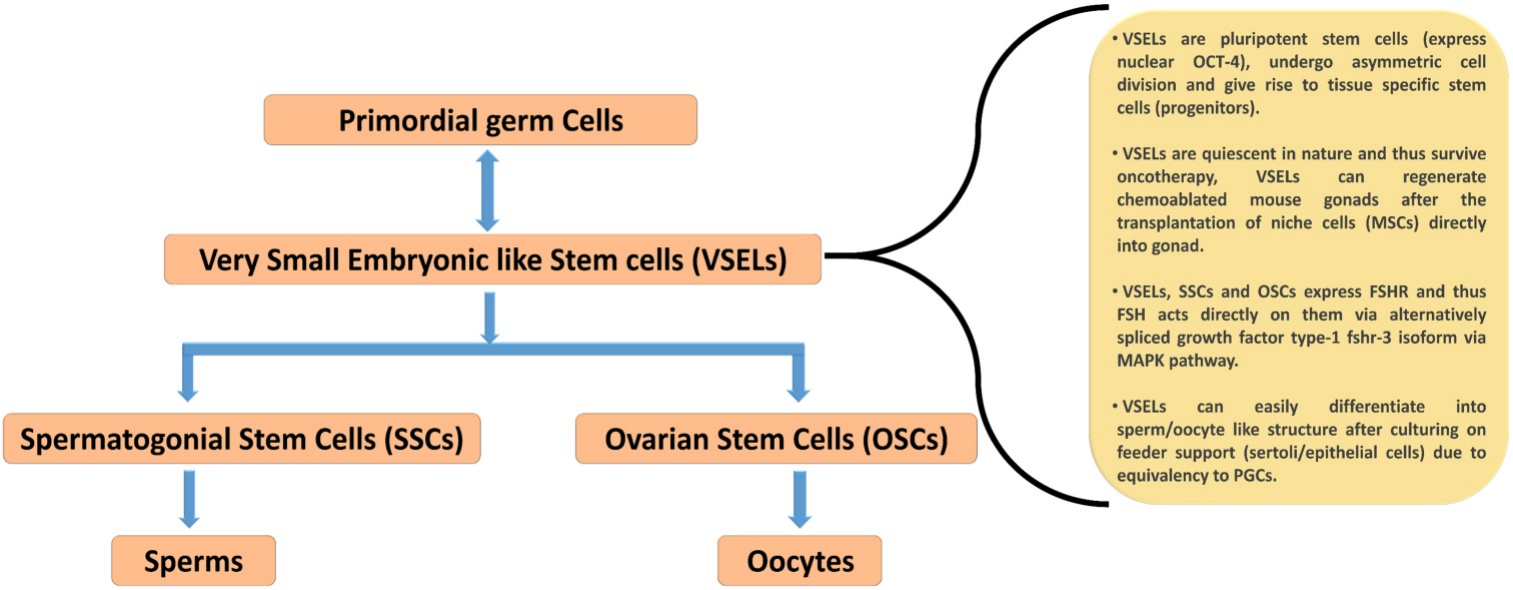
** Schematic diagram representing the hierarchy of stem cells found in mammalian gonad and their prominent characteristics.** VSELs are considered as developmentally equivalent to primordial germ cells due to their spontaneous differentiation into oocytes and sperm like structure during *in vitro* culture condition. VSELs are quiescent small sized stem cells, exist in gonads and give rise to a marginally bigger SSCs/OSCs that further differentiate into gametes by undergoing symmetrical cell divisions (according to the hypothesis put forward by Bhartiya et. al. (43). Re-drawn from Figure 6.2 of Bhartiya et al. 43.

Similar to VSELs reported in adult human ovaries, these cells have also been documented in the testis of humans and mice 41,70,71. The presence of VSELs in the testis was first reported by Ratajczak's group 72. VSELs in the testis exist as a sub-population among the well-examined SSCs near the basal region of the seminiferous tubule. These were characterized *in vivo* using mouse testicular section based on the nuclear OCT-4 immunolocalization 41,73. OCT-4 has two major isoforms namely OCT-4A (that reflect pluripotent state), and OCT-4B (that reflect differentiated state) 41,70. VSELs are pluripotent in nature and express pluripotent nuclear OCT-4A in the nucleus, whereas SSCs are found to express cytoplasmic OCT-4B 43. The expression pattern of FSHR in mouse testicular VSELs and SSCs and the impact of FSH treatment on their stimulation have been recently examined using mice model *in vivo* 41. Testicular VSELs were studied through flow cytometry and were found to be positive for stem cell antigen (SCA) and negative for lineage (LIN) and hematopoietic marker (CD45), thus characterized as [LIN^-^/CD45^-^/SCA-1^+^]. FSH plays a crucial role in spermatogenesis and it has been confirmed that both VSELs and SSCs express FSHR, and further they respond to FSH treatment. According to this study, pluripotent VSELs are the most primitive stem cells in the testis and FSH acts directly on them through FSHR3, not through canonical FSHR1, to stimulate their asymmetric cell division to undergo self-renewal. These VSELs then further develop into SSCs through symmetric cell division, undergo clonal expansion, and eventually terminally differentiate into sperm. Consistent with these observations, a similar positive impact of FSH on sheep testicular stem cell stimulation was also observed by Bhartiya and her group 70. VSELs can survive chemotherapy and their surge was found after treatment with FSH (0.045 ± 0.008% control Vs 0.1 ± 0.03% FSH treatment). Additionally, it was also reported that VSELs may effectively restore spermatogenesis after the transplantation of cells that involve stem cell niche formation (i.e. Sertoli cells and/or bone marrow mesenchymal stem cells) 70. Recently, James *et al.* 73 determined the presence of OCT-4A positive VSELs in the endometrial region of adult mouse uterus. These cells were also found to express various isoforms of FSH. Taken together, these studies clearly demonstrate that FSHRs are not exclusively expressed on granulosa and Sertoli cells, but on uterine stem cells to stimulate their self-renewal and proliferation as in testis and ovary, suggesting that FSH/FSHR system possibly plays a crucial role in reviving the gonadal stem cell population (which were believed to be non-existence). A schematic summarizing the mainpoints of these recent studies is depicted in Figure 4.

### Function of FSH-FSHR in cancer and cancer stem cells

Cancer stem cells drive the cancer progression through modulating signalling pathways and regulating miRNAs to offer therapy resistance and promote metastasis 74-76. FSH plays a significant role in facilitating this role of CSCs in malignancy. FSH has been reported to exhibit anti-apoptotic effect in OSCs by modulating stem cell signalling pathway 77. Besides, FSH also promotes proliferation of OSCs by activating sphingosine kinase 78. As reported above, both VSELs (with nuclear OCT-4) and OSCs (with nuclear OCT-4) are part of the ovarian stem cell population. OSCs are equivalent to SSCs in the testes and can spontaneously differentiate. OSCs express FSH and are thus responsive to FSH-FSHR signaling leading to: self-renewal, clonal expansion, neo-oogenesis, and primordial follicle assembly. Although VSELs exhibit stemness similar to that of embryonic carcinoma cells, they are relatively quiescent 79. Nevertheless, chemoresistant VSELs could undergo oogenesis in mice in response to FSH. These findings warrant the investigation and generation of novel onco-fertility management strategies 39. Accumulating body of evidence demonstrates that FSHR is expressed in different types of tumors, such as: prostate, ovarian, thyroid, neuroendocrine, pancreatic, pituitary, and soft tissue sarcomas 80. FSHR3 signaling promotes the proliferation of ovarian cancer cells 35, implying the role of FSH-FSHR signaling in tumorigenesis and identifying this pathway as a potential anti-tumorigenic therapeutic target. The occurrence of testicular germ cell tumors (TGCTs) due to developmental exposure to endocrine receptors is attributed to epigenetic alteration in VSELs, driving enhanced proliferation and compromised differentiation 81.

The major transcriptional regulatory pathways controlling stem cell signaling, cellular reprogramming that are responsible for cellular pluripotency are: Hedgehog, Ephrin, WNT, and certain microRNA pathways 82. The core transcriptional factors of these pathways are a part of a complex protein-protein interaction-dependent network that maintains stem cell pluripotency by modulating their own expression to upregulate pro-pluripotency genes and inhibit pro-differentiation of genes. This transcription regulatory network is an effector matrix responsive to several kinase signal transduction pathways activated by intrinsic and extrinsic stimuli. OCT-4, a stem cell marker, is overexpressed in several types of human cancer and can induce resistance to chemotherapy and inhibition of apoptosis.

FSH-FSHR upregulates OCT4 expression in ovarian cancer stem cells, most likely through its regulation of GSK3β/β-catenin and PI3K/Akt effector pathways 77,83. Overexpression of OCT4 results in increased levels of Notch, Sox2, and Nanog, leading to expansion of CD44^+^/CD117^+^ cells exhibiting stemness 77. Moreover, FSH-FSHR-mediated upregulation of OCT4 and SNAIL through the ERK1/2 pathway results in epithelial-mesenchymal transition and invasion in epithelial ovarian cancer 84. Further, FSH modulates ovarian stem cells through FSHR3 to stimulate potential self-renewal and clonal expansion 85. The formation of cystic ovarian follicles depends on results from increased levels of LH, and FSH signaling evident from the absence of follicles in transgenic mice in which FSHβ or FSHR genes are knocked out 86. Therefore, proper functioning of the FSH-FSHR system is imperative for maintenance of gonadal stem cells in an effort to prevent them from triggering carcinogenesis. This regulation is exerted through various signaling cascades under the control of FSH. Notably, by stimulating phosphorylation of sphingosine kinase 1 (SphK 1) and 2 (SphK 2), FSH induces proliferation of ovarian cancer cells in epithelial ovarian cancer 78.

Polymorphisms in the FSHR gene have been found to modulate the risk of testicular germ cell tumor formation. Polymorphisms in the coding region, such as: Ala^307^Thr, and Ser^680^Asn in exon 10 of FSHR gene alone or in combination with polymorphisms in the promoter region (-114 T/C and -29 G/A) are reported to be associated with a reduced risk of the disease 87. In contrast, homozygous Ala^307^/Ser^680^ alleles have been observed to increase the risk and recurrence of ovarian cancer 88. Although the risk associated with different types of gonadal cancer with FSHR polymorphisms are unknown, therefore underlying mechanisms employed by these polymorphisms in modulating FSHR function require further explorations.

### FSH-FSHR signaling in malignant tumors

A few, but conflicting reports are available regarding the FSH-FSHR signaling system pertaining to tumor malignancies 89,90. However, a notable exception exists in such research efforts relating to ovarian cancer, for which a large body of evidence exists documenting the role of this system in disease pathogenesis 91-94. FSH reduces the expressions of PDCD6 (programmed cell death gene 6) and DR5 (death receptor 5). As a consequence of this process, it is likely that apoptosis of epithelial ovarian cancer cells is inhibited. This observation is linked to the loss-of-function in the FSH-FSHR signaling during disease progression 95. In contrast, in the case of serous ovarian cystadenocarcinoma, FSH augments the PI3K/AKT pathway and increases VEGF expression 96. Likewise, FSH is involved in Gankyrin-mediated ovarian oncogenesis through the PI3K/AKT/hypoxia-inducible factor (HIF)-1α/cyclin D1 pathway 97. FSHR-3 expression orchestrates the mitogenic and cellular proliferative activities of FSH, and is associated with epithelial ovarian cancer 36. Overexpression of the receptor due to its association with: increased c-Myc, human epidermal growth factor receptor 2 (HER-2)/neu, and EGFR, as well as a reduction in prohibitin and RIIb, facilitates the increase in cell proliferation and induces a concomitant aggressiveness of the carcinoma 94. In this contest, several studies have shown an association of FSH-FSHR signaling with both a VEGF-dependent and -independent manner in promotion of angiogenesis and the consequent cell proliferation and migration of ovarian cancer 98,99.

### Recent updates for FSHR expression in different malignancies

As mentioned above, the involvement of FSH-FSHR signaling in malignancies in tissues other than gonadal cancers is limited. However, gradually accumulating evidence points towards the involvement of FSH-FSHR signaling in cancers of various tissues other than gonads. In this regard, a recent trial reporting FSHR expression in vascular endothelial cells in prostate, breast, and urothelium cancers are reported 100. Studies on thyroid cancer suggest FSHR as a marker for malignancy 101,102. Augmented FSHR expression is also observed in pituitary adenomas, adrenal tumors, and pancreatic neuroendocrine tumors 103,104. Major studies validating FSHR expression in different types of cancers are presented in Table 1.

### Effector pathways: events and outcomes of FSH-FSHR activation

#### FSH-FSHR activated cytosolic signaling pathways

**Figure 5:**
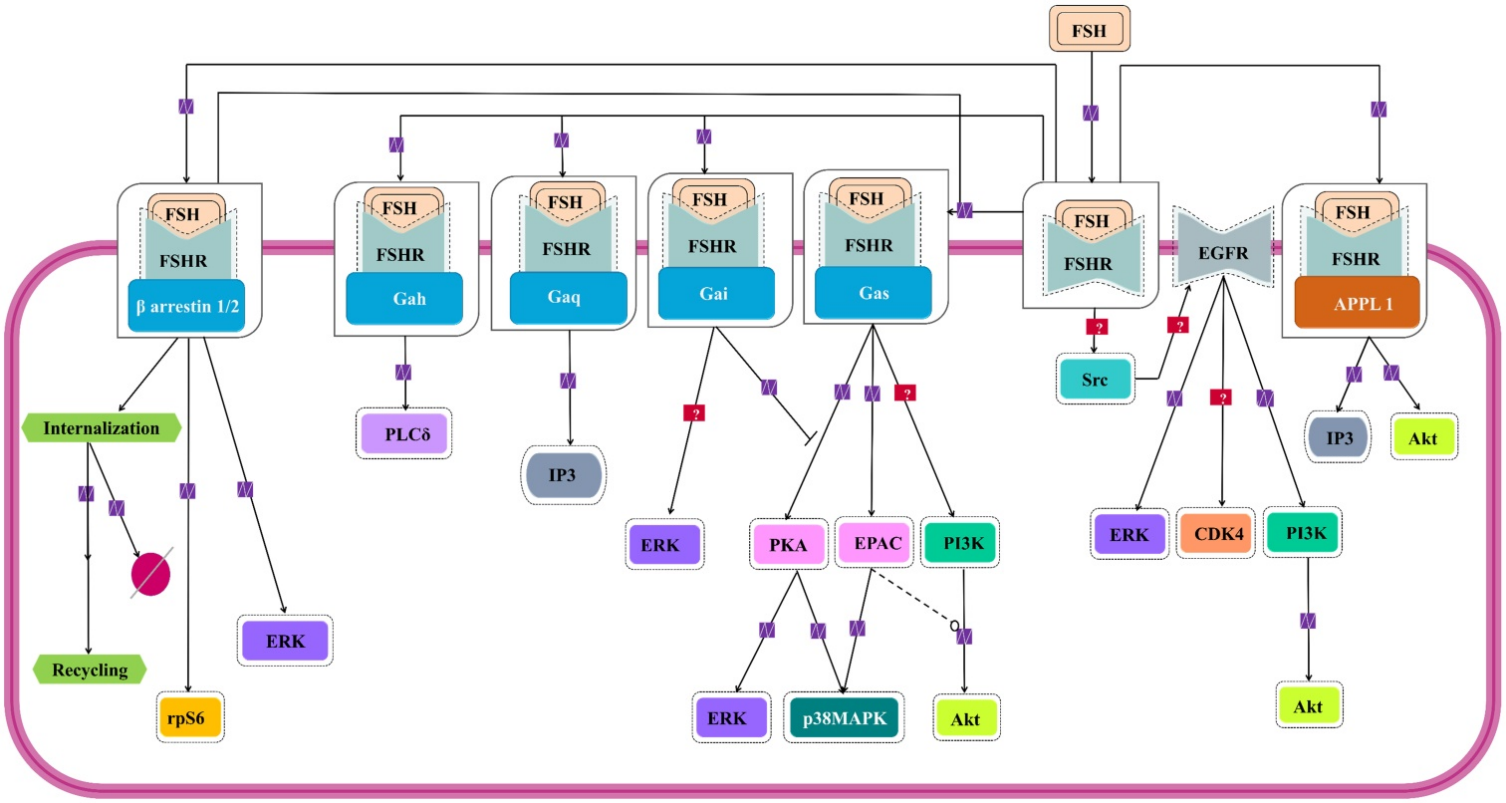
** Signaling cascades triggered by FSH-FHSR system in target cells.** Besides the main Gαs/cAMP/protein kinase A (PKA) pathway that has been believed to be the mainstay of FSH signal transduction within the target cells, accumulating evidences over the last decade show that FSHR can also engage APPL1-mediated signaling, EGFR transactivation and β-arrestin-dependent pathways. Solid lines represent facilitatory outcomes whereas, the dotted ones pertain to the inhibitory results. Adapted from Bloaguen et al. 111 under CC-BY License*.*

For almost over two decades, FSH-FSHR has been believed to be acting through the Gαs/cAMP/PKA signaling pathway to mediate its biological actions in the target cells 109,110. Interestingly, evidences show that FSH-FSHR system works through other pathways as well, and curiously, some of them are G- protein independent. Figure 5 schematically summarizes the G protein-dependent and -independent signaling pathways that gets activated within the target cells by FSH-FSHR system.

#### The Canonical Gαs-dependent Pathway

**Figure 6:**
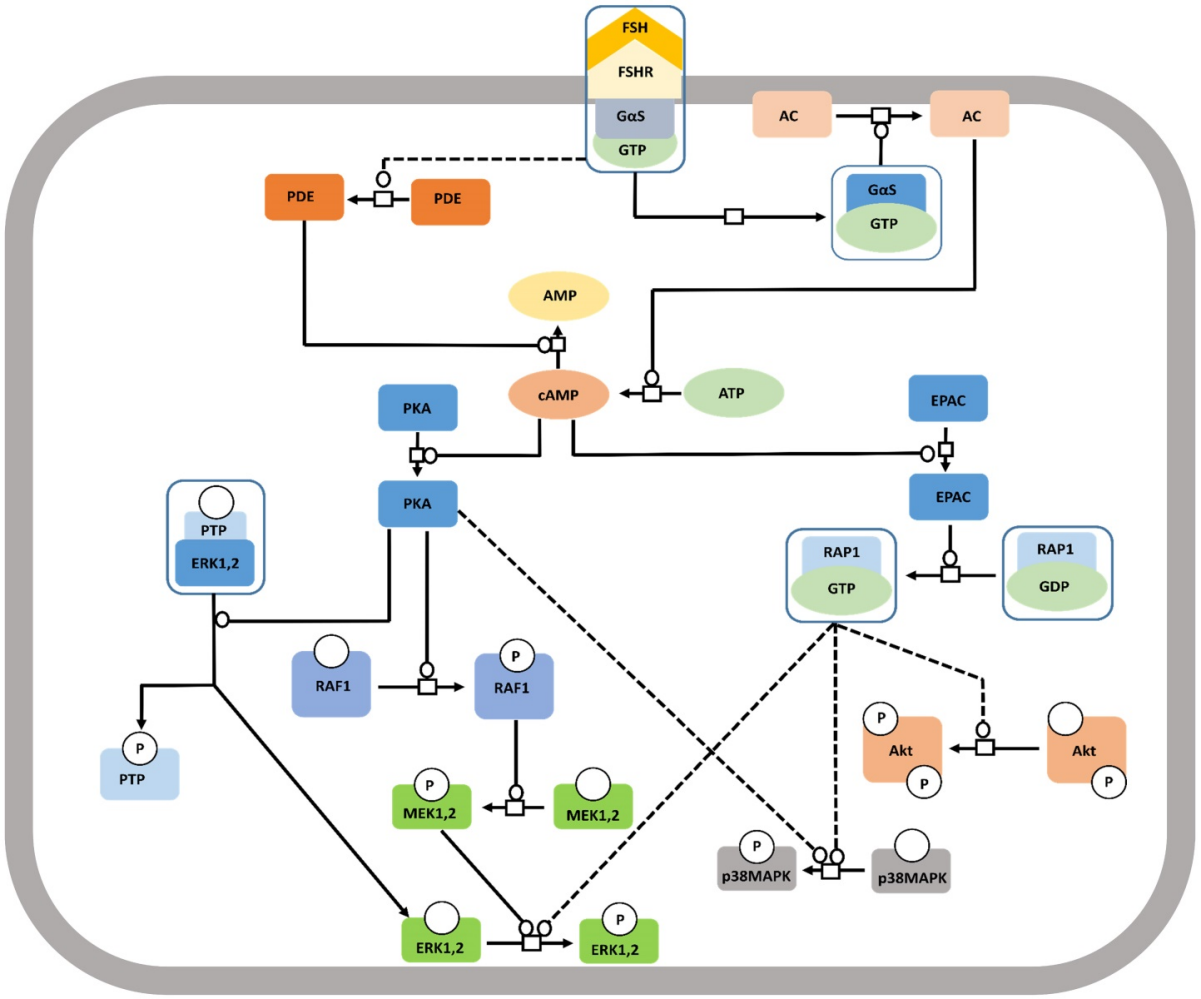
** Canonical Gαs/cAMP/PKA pathway at FSHR in target cells.** The Gαs/cAMP/PKA pathway has been the most studied and is associated with various intracellular events. It is now acknowledged that PKA is not the sole target of cAMP accumulation. Indeed, EPAC is also activated upon FSH stimulation. Both PKA and EPAC contribute to the activation of MAPK, ERK and p38, whereas EPAC also leads to Akt activation. Solid lines represent facilitatory outcomes whereas, the dotted ones pertain to the inhibitory results. Adapted from Gloaguen et al. 111 under CC-By License*.*

The well-studied Gαs pathway shows that FSH-FSHR interactions lead to coupling of the receptor to Gαs subunit inducing adenylate cyclase activity to produce cAMP 112. cAMP, in turn, activates the downstream effectors like protein kinase A (PKA) and EPAC (exchange protein directly activated by cAMP). Binding of cAMP to PKA releases the catalytic subunits of the latter, that phosphorylates several targets, both cytosolic and nuclear 113, 114. FSH mediated activation of cytosolic ERK-MAPK pathway is believed to be PKA dependent 115,116. PKA-mediated phosphorylation of the protein phosphatase (PTP) leads to disruption of the PTP-ERK complex resulting in increased ERK phosphorylation given that MEK is constitutively phosphorylated 117. Along another pathway, PKA activates Raf1, thereby, inducing MEK followed by ERK 118,119. FSH induced MAPK p38 phosphorylation is also PKA dependent 120,121. As a downstream effector of FSH-FSHR activation, EPAC binds accumulated cAMP to trigger GDP to GTP exchange on Rap1, and its consequent, activation in the granulosa and surface epithelial cells of ovary 23,121. Activated Rap1 consequently, actuates ERK, p38 and Akt 23,122. The pathway has been depicted schematically in Figure 6.

#### β-arrestin-dependent Pathway

**Figure 7:**
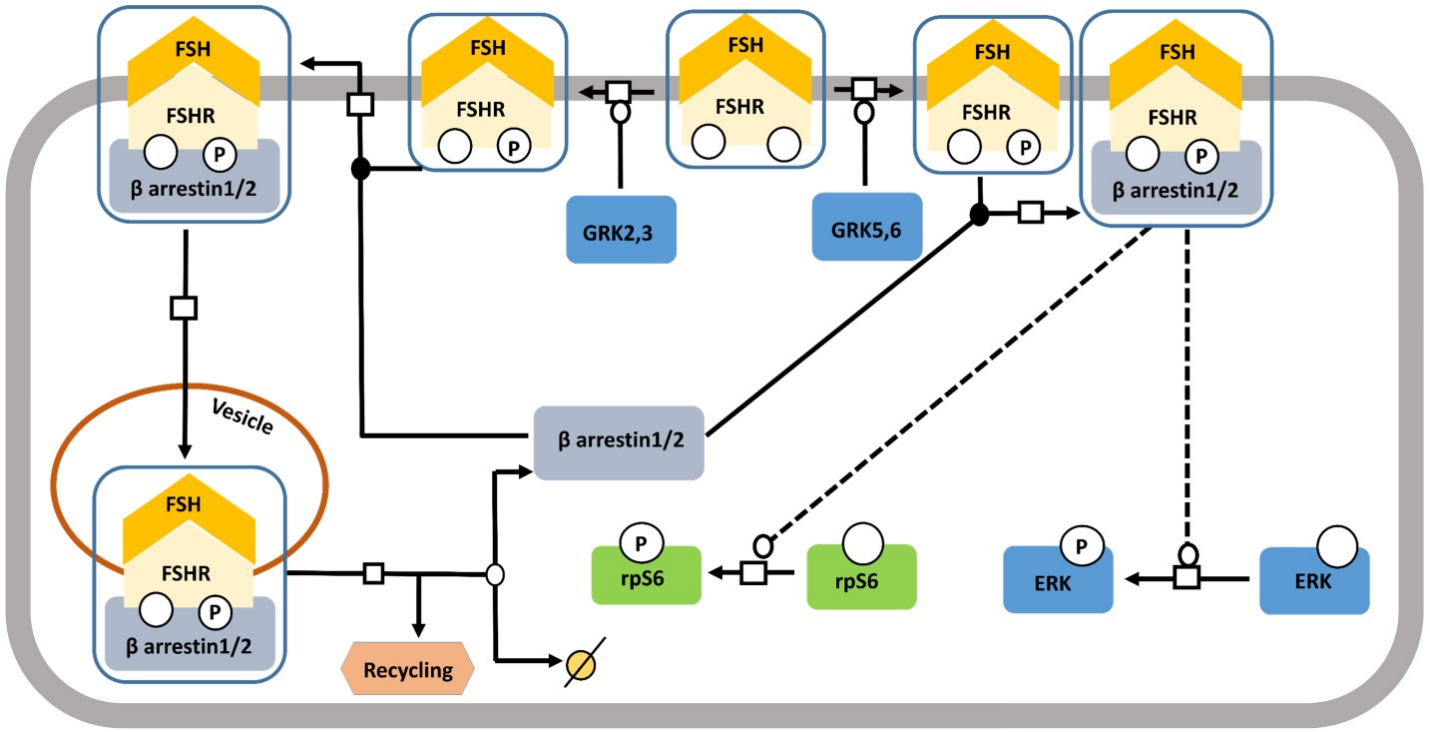
** FSH- induced β-arrestin-dependent pathway. Initially, the perception of β-arrestins' role was restricted to the control of desensitization of FSHR and its recycling.** This view has progressively evolved towards a more general role of β-arrestins as adapters and transducers leading to the activation of MAPK, ERK and rpS6 independent of G proteins upon FSH stimulation. GRK2/3 and GRK 5/6 control the fate of the activated FSHR (i.e., desensitization or signaling) presumably through phosphorylation of distinct serine and threonines within the receptor's C-tail. Solid lines represent facilitatory outcomes whereas, the dotted ones pertain to the inhibitory results. Adapted from Gloaguen et al. 111 under CC-BY License.

Starting off as a pathway for desensitizing and recycling FSHR, β-arrestin pathway, gradually received the recognition of one with adapters and transducers of FSH-FSHR signaling 123 (Figure 7). It has been shown in different models that binding of FSH to FSHR gets phosphorylated by G protein-coupled receptor kinases (GRKs) 2, 3, 5, and 6 116,124-127, at the serine-threonine clusters in C-terminal intracellular domain, particularly by GRK 2 116. GRK 5 and 6 mediated FSHR phosphorylation evoked responses similar to those found with GRK 2, albeit to a lesser extent 116. β-arrestin mediated receptor recycling elicits G protein independent transduction of signals, not only, through FSHR, but also through several other 7TMRs 116,123,128,129. Interestingly, unlike, the rapid, transient nature of G protein mediated transduced signals, the ones being cascaded through β-arrestin pathway are gradual and sustained 116. Besides, β-arrestin mediate rpS6 phosphorylation in HEK293 cells in response to FSHR activation 130.

#### PI3K/mTOR pathway

**Figure 8:**
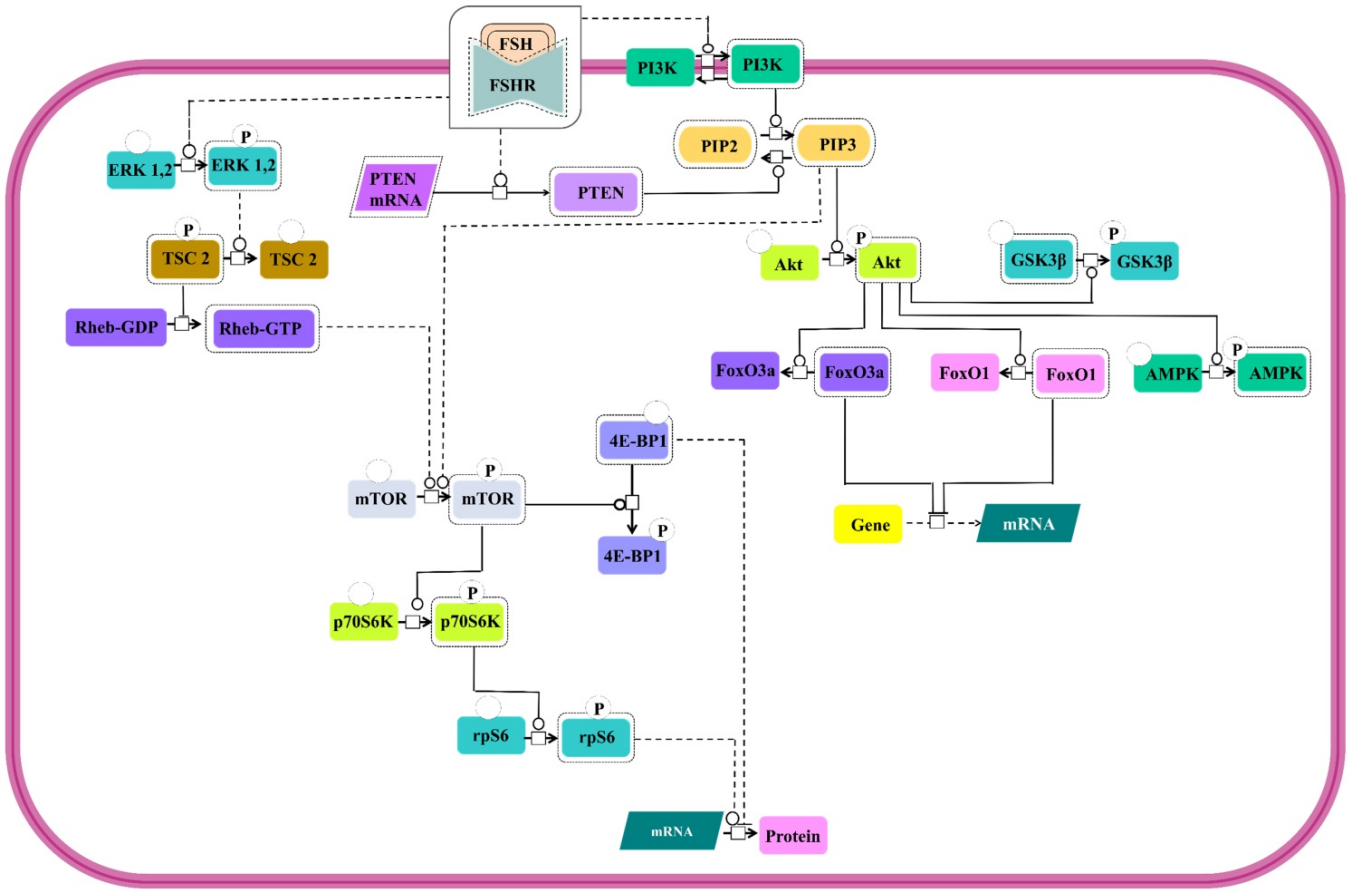
** PI3K/mTOR signaling at FSHR in target cells.** The PI3K/mTOR pathway plays an important role in FSH-induced actions, including proliferation, regulation of gene expression as well as protein translation. Solid lines represent facilitatory outcomes whereas, the dotted ones pertain to the inhibitory results. Adapted from Gloaguen et al. 111 Under CC-BY License.

Involvement of PI3K/mTOR pathway as an effector route for FSH-FSHR mediated signaling has been substantiated through several studies. All the pathways show PI3K activation and PIP3 accumulation 131-133 and several of downstream signal cascades that involves Akt activation 24,28,131,133-139 leading to phosphorylation and deactivation of GSK3β 28,140 and AMPK 141 and inactivation of transcription factors, FoxO3a and FoxO1 133,134,136,138,139,142 (Figure 8). However, in a contradictory report, PTEN, the negative regulator of PIP3 accumulation, has been noted to be upregulated by FSH stimulation, thus, preventing FSH-mediated cell proliferation 133. In addition to PI3K/PIP3 pathway inducing the mTOR phosphorylation 132,138, FSH-FSHR activated ERK pathway also triggers mTOR signaling via lifting of negative regulation on Rheb through TSC2 phosphorylation 134,143,144. Activated mTOR pathway ensures p70S6K activity 132,133,138,143,144 resulting in phosphorylation of rpS6 132,134,144. Active mTOR phosphorylates to inactivate 4E-BP1, the negative regulator of protein translation 134. Simultaneous rpS6 activation and 4E-BP1 inactivation indicate that FSH-FSHR stimulation also modulates effector molecules at the level of protein translation, besides, influencing transcription.

#### FSH controlled nuclear events

**Figure 9:**
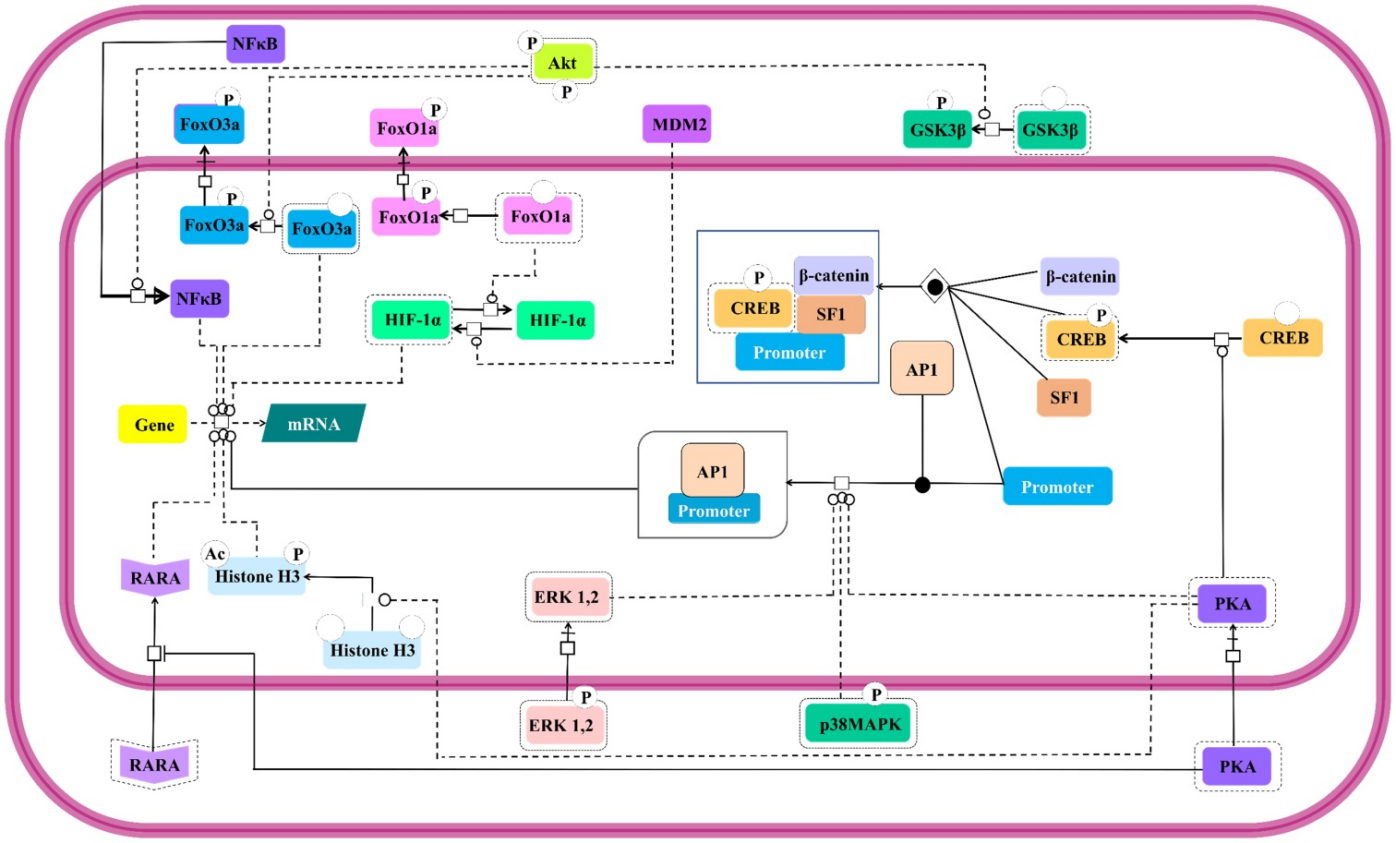
** Nuclear events controlled by FSH. Gene transcription has long been known to be affected by FSH.** Multiple signaling pathways that are activated upon stimulation (i.e., PKA, p38, ERK and Akt) subsequently trigger the activation or suppression of the activities of various transcription factors within the nucleus. Solid lines represent facilitatory outcomes whereas, the dotted ones pertain to the inhibitory results. Adapted from Gloaguen et. al. 111 under CC-BY License.

Modulation of expressions of genes involved in steroidogenesis by FSH is a well-established 145. Besides, it is also known to control expressions of genes involved in cell cycle through the regulation of Smad proteins 146. This FSH mediated regulation of nuclear events is as complex as its cytosolic effector signal cascading. Interestingly, the activated cytosolic effector molecules undergo nuclear translocation to take the signal transduction triggered by FSH-FSHR stimulation across the nucleus leading to gene expression. Catalytic subunit of FSH-activated PKA translocates to nucleus to set off CREB by phosphorylating it on S133, thus, regulating the expressions of the genes containing cAMP response element (CRE) in their upstream region 117,140. Nuclear PKA also facilitates the recruitment of Activator Protein-1 (AP-1) at its cognate binding regions upstream of the open reading frames 147. PKA extends its effect beyond modulation of promoters to post-translational histone modifications, like, histone H3 phosphorylation and acetylation 148 favoring cell division 149. In an indirect *modus operandi* to modulate gene expression, cytosolic PKA binds to retinoic acid receptor alpha to inhibit its nuclear translocation, thereby, modulating the expressions of the genes under the regulation of this transcription factor 150. Akt also affects gene expression in PKA independent manner. As mentioned earlier, activated Akt phosphorylates the transcription factors, FoxO1a and FoxO3a resulting in their nuclear exclusion and concomitant MDM2-dependent HIF-1α activation and repression of pro-apoptotic genes, respectively 28,131,136,138,139. Akt mediated GSK3β deactivation indirectly supports LEF dependent transcription 24,140. Akt also promotes nuclear translocation of NFκB 151. Likewise, activated MAP kinases, ERK and p38 are reported to affect transcriptional activities regulated by AP-1 and CREB 152. All these nuclear events which are affected by FSH-FSHR stimulation are depicted in Figure 9.

#### FSHR interacting proteins

**Figure 10:**
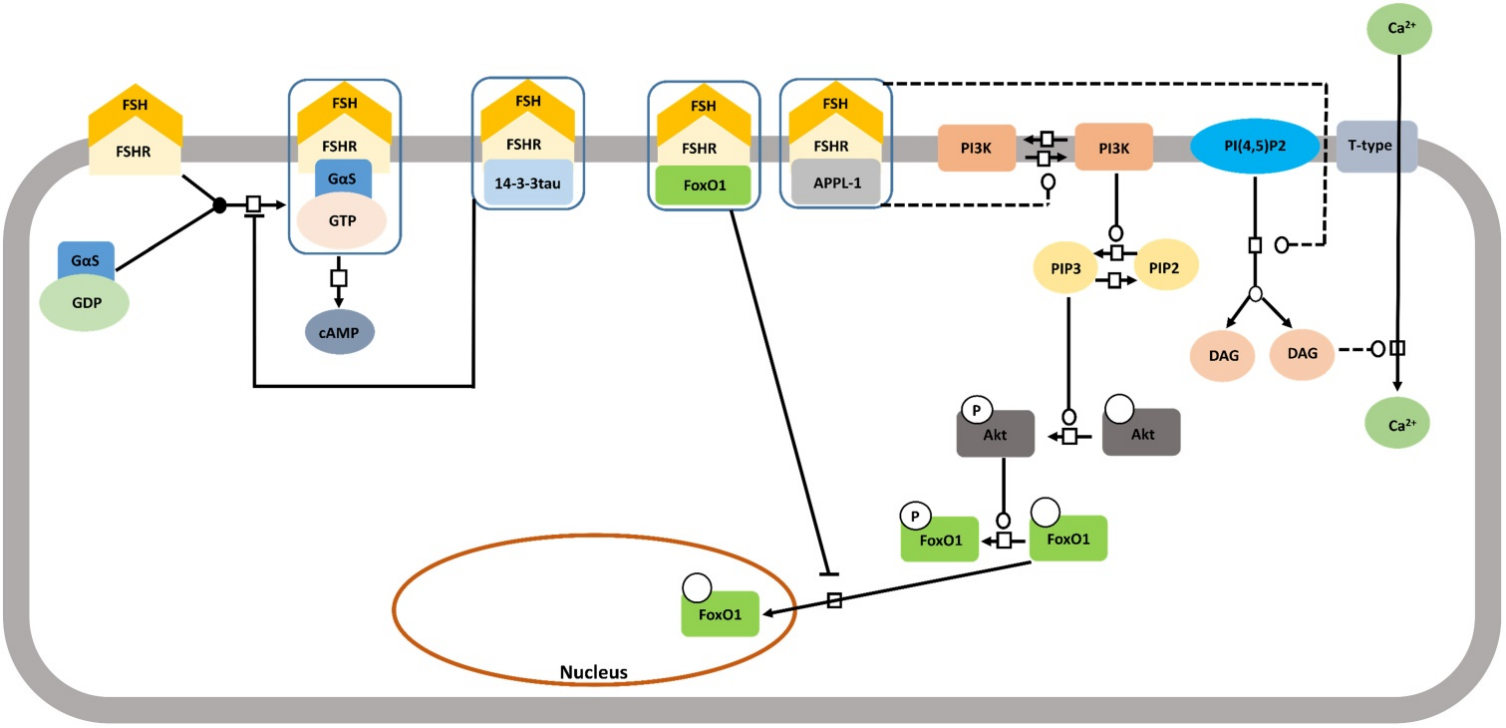
** Proteins interacting with FSHR thus impacting the signalling pathway.** A number of proteins interact with FSHR thus affecting FSH-induced signaling pathways. These include 14-3-3tau, FoxO1 and APPL1, the latter two being involved in the control of the PI3K/Akt pathway downstream of FSHR. Solid lines represent facilitatory outcomes whereas, the dotted ones pertain to the inhibitory results. Adapted from Gloaguen et al. 111 under CC-BY License.

Until now we elaborately described about the pathways that get activated mostly through second messengers due to the interaction between FSH and FSHR. FSHR being a member of the GPCR family, this mode of activating signaling cascade is expected. Interestingly, FSH-FSHR interaction can also adopt alternative pathways to transduce signals and this largely, involves direct physical interaction of certain proteins with FSHR (Figure 10). A number of such interacting proteins have been identified and shown to influence FSH-mediated intracellular signaling 153. APPL1 is one such protein found to interact with the first and second intracellular loops of FSHR, concomitantly, mediating PI3K and Akt activation 136. Residues K376, L377 and F382 in the first intracellular loop of FSHR are involved in the interaction with APPL1, with K376 being particularly, indispensable for linking activated FSHR to the inositol-phosphatase pathway and intracellular calcium mobilization 154. Although, several studies link APPL1 with FSH triggered intra-cellular calcium accumulation, yet, the extent to which this association is required remains yet to be deciphered 155-158. APPL1-FSHR interaction is also associated with nuclear exclusion of FoxO1a through Akt-dependent phosphorylation 159. Along another pathway, FoxO1a is reported to exhibit direct interaction with FSHR, which prevent its nuclear translocation. Scaffolding protein 14-3-3τ is yet another factor known to interact with the second intracellular loop of FSHR at the ERW motif. The 14-3-3τ is suspected to compete with Gαs binding at the spot based on the reported observation that overexpression of the scaffold protein reduced FSH-induced cAMP accumulation 159,160.

### Potential therapeutic implication of FSH-FSHR system

The association of identified mutations in FSHR with specific cellular functional has enabled this information to be utilized in identifying therapeutic targets and designing of novel therapeutics. For example, pharmacophore (Org 41841) was designed to bind FSHR at a site other than that used by FSH and improve the membrane localization of the receptor in the presence of an Ala^189^Val mutation, known for misrouting the receptor and retaining it in the endoplasmic reticulum 161. Such studies hold promise for identifying treatment modalities for reproductive abnormalities owing to non-functioning or dysfunctional FSHR. Similar studies to allow a better assessment of the potential of FSH-FSHR system as therapeutic targets have been conducted. For example, a study validating the expression of FSHR in renal carcinoma cells in response to sunitinib 108. FSH-FSHR signaling promotes angiogenesis. Degarelix, a gonadotropin-releasing hormone receptor antagonist, was used to block FSH production in a multi-treated metastatic colon cancer patient with encouraging results 162. Urbanska and colleagues evaluated a T cell-based immunotherapy targeting FSHR with promising results that warrant further investigations 163. In addition, small inhibitory RNA- (siRNA) mediated modulation of the FSH-FSHR system is also being explored. *In vitro* observations revealed a possibility of slowing the growth of FSH-responsive tumors through siRNA-mediated silencing of FSHR3 in mouse ovarian cancer cell lines 164. Taken together, these evidences show that the FSH-FSHR could be a very effective anti-cancer target.

### Future directions in modulating FSH-FSHR signaling

The elevated FSH levels of ovarian cancer corroborate with the increased expression of FSHR in several cancers. Upregulated endothelial FSHR levels in cancers are associated with vascular remodeling and tumor angiogenesis, whereas, its epithelial counterpart facilitates cell proliferation, migration and invasion 165. Therefore, FSHR has emerged as a potential anti-cancer therapeutic target and prognostic tool. However, to the best of our knowledge no clinical trial has been performed for the clinical application of FSH-FSHR in treatment of cancer. However, there are several clinical trials have been performed or underway for the application of stem cells to treat different types of cancers 166. A number of small non-peptide ligands, such as: thiazolidinediones, benzamides, and dihydropyridine Org 24444-0, have also been explored for their effects on FSHR-induced signaling 167-169. In contrast, non-peptide ligands, such as tetrahydroquinolines, antagonized cAMP production without affecting FSH-FSHR interactions 170 have been explored. Single chain analogs of FSH have also been developed to ameliorate the effects of inactivating mutations of the FSH-FSHR signaling system 171. However, these compounds still need to be tested in clinical trials. In addition, antibody-mediated targeting of FSH-FSHR system is another actively explored avenue for modulating this FSH/FSHR signaling system 172-174. FSH/FSHR antagonists in the form of nanobodies are also being explored as potential modulators of this signaling system 175. Last, but not the least are the pepducins 176 and aptamers 177, two classes of pharmacological modulators, which are based on their performance as GPCR modulators, deserve thorough evaluation in the context of FSH-FSHR signaling. Altogether, these evidences show that modulation of FSH-FSHR signalling for anti-cancer therapeutic outcomes are past the speculative stage and warrants for in-detail investigations for being developed into effective treatment options.

## Summary

FSH/FSHR signaling controls several crucial biological pathways, therefore, malfunctioning of this signaling system is bound to culminate into several disorders, including cancer. This review mainly covers the connection between FSH and FSHR signaling and its role in cancer. Attempts were made to provide a comprehensive understanding of the various aspects under the control of the FSH/FSHR signaling system. This included the effect on stem cell maintenance and propagation, as well as sources of variations in the functioning of FSH or FSHR (or both). Studies reporting various mutations, polymorphisms, and alternatively spliced variants of FSHR have correlated these products with their respective phenotypes, clearly suggesting that such genetic alterations may result in infertility. Discussion on *in vitro* functional studies provides valuable information on FSH-FSHR interactions at the molecular level. Few mutations have been reported in either subunit of FSH and may lead to pathological implications. Comparatively, FSHR has a greater number of mutations, some resulting in gain-of-function, while others resulting in a loss-of-function. These mutations affect hormone binding ability or hormone-induced signaling ability by introducing structural changes in the receptor. Various polymorphisms of FSHR have been associated with risks of different types of cancers. Finally, the therapeutic implications of this information have been documented with a positive future perspective on its potential applications.

Many aspects of the FSH-FSHR signaling system require better insight and clarifications, for which detailed studies are needed. Moreover, along with the understanding of the alterations in the signaling mechanisms that affect the prognosis of cancer, it is important to devise protocols of translating this foundational knowledge to clinical applications. This review has put together several aspects of FSH-FSHR signaling which are highly informative and provide quick reference to facilitate the designing of elaborate studies with the objectives dedicated towards harnessing the therapeutic aspects and potentials of FSH in fertility and disorders especially cancer.

## Figures and Tables

**Table 1:** Major studies validating expression of FSHR in different types of tumor tissues

Type of Tumor (No. of Patient)	Type of study, Aim	Results	Reference
Prostate (773), Breast (112), Urothelial (77), Pancreas (67), Kidney (64), Colon (15), Hepatocellular (15), Lung (15), Testicular (8), Gastric (6), Ovarian (6)	Retrospective, expression of FSHR in different tumors	Highly selective expression of FSHR on the surface of the blood vessels (endothelium) of all tumors (uniform expression of FSHR in all tumors with specific pattern, no expression in normal and inflammatory issues)	100
Ovarian (156)	Prospective, correlation of FSHR expression and overall survival	Tumors overexpressing FSHR: decreased overall survival	105
Ovarian (153)	Retrospective, correlation of HER-2 and FSHR expression and overall survival	HER-2 expression related to an adverse impact on overall survival, only in non FSHR expression (small sample size of HER-2 expression	106
Prostate (76), Lung (46), Breast (42), Colon (34), Kidney (5)	Retrospective, expression of FSHR in different tumors, primary/metastatic sites	FSHR is expressed by the endothelium of blood vessels in the majority of metastatic tumors (no specific pattern of expression identified between primary and metastatic site, no expression identified in normal and inflammatory issues)	107
Kidney (50)	Prospective, correlation of FSHR expression with sunitinib therapy	FSHR-positive stained vessels were on average 5- and 8-fold higher in responders and patients with stable disease, respectively, than in nonresponders	108
